# Associations between air pollution and markers of neuroinflammation, synaptic dysfunction and core Alzheimer’s disease pathology vary by *APOE* genotype

**DOI:** 10.1007/s12640-026-00786-2

**Published:** 2026-04-07

**Authors:** Kana Kimura, Ira Driscoll, Noah Cook, Sonia Shahzad, Tobey J. Betthauser, Sterling C. Johnson, Sanjay Asthana, Catherine L. Gallagher, Bruce P. Hermann, Mark A. Sager, Kaj Blennow, Henrik Zetterberg, Cynthia M. Carlsson, Gwendlyn Kollmorgen, Ozioma C. Okonkwo

**Affiliations:** 1 Department of Psychology, University of Wisconsin – Milwaukee, Milwaukee, WI 53711 USA; 2 Wisconsin Alzheimer’s Disease Research Center, Department of Medicine, School of Medicine and Public Health, University of Wisconsin-Madison, Madison, WI USA; 3 NeuroGenomics and Informatics Center, Washington University School of Medicine, St. Louis, MO USA; 4 Wisconsin Alzheimer’s Institute, Madison, WI USA; 5 Geriatric Research Education and Clinical Center William S. Middleton VA Hospital, Madison, WI USA; 6 Department of Neurology, University of Wisconsin School of Medicine and Public Health Madison, Madison, WI USA; 7 Department of Psychiatry and Neurochemistry Institute of Neuroscience and Physiology Sahlgrenska Academy, University of Gothenburg, Mölndal, Sweden; 8 Clinical Neurochemistry Laboratory Sahlgrenska University Hospital, Mölndal, Sweden; 9 Pitié-Salpêtrière Hospital, Paris Brain Institute, ICM, Sorbonne University, Paris, France; 10 Neurodegenerative Disorder Research Center, Division of Life Sciences and Medicine, Department of Neurology, Institute on Aging and Brain Disorders, University of Science and Technology of China and First Affiliated Hospital of USTC, Hefei, P.R. China; 11 Department of Neurodegenerative Disease, UCL Institute of Neurology, Queen Square London, UK; 12 Dementia Research Institute at UCL, London, UK; 13 Hong Kong Center for Neurodegenerative Diseases, Clear Water Bay, Hong Kong, China; 14 Department of Pathology and Laboratory Medicine, School of Medicine and Public Health, University of Wisconsin, Madison, WI USA; 15 Centre for Brain Research, Indian Institute of Science, Bangalore, India; 16 Roche Diagnostics GmbH, Penzberg, Germany

**Keywords:** Air pollution, PM₂.₅, *APOE* ε4 allele, Alzheimer’s disease, AD biomarkers, Neuroinflammation, Gene-environment interaction

## Abstract

To determine whether long-term residential air pollution [AP; ozone (O₃) and fine particulate matter (PM₂.₅)] is associated with (1) incident mild cognitive impairment (MCI) or Alzheimer’s disease (AD), (2) biomarkers of core and AD-relevant pathology, and (3) whether these relationships are moderated by APOE4+/- (carrier/non-carrier of one or both ε4 alleles) status or mediated by neuroinflammation. Sample included 795 participants (Mage 68.7 ± 7.9; 68% female) from the Wisconsin Alzheimer’s Disease Research Center and Wisconsin Registry for Alzheimer’s Prevention parent studies, both enriched for AD risk at enrollment based on parental AD history. Residential zip code and 2009–2021 EPA-based annual AP reports were used to estimate individual exposure. Cox proportional hazards models assessed MCI/AD risk. Linear regressions examined the relationships between AP exposure and biomarkers of core and AD-relevant pathology, with and without APOE4 + stratification. Causal mediation analysis examined whether markers of inflammation mediated the AP-AD pathology relationships. Neither O₃ nor PM₂.₅ exposure predicted MCI/AD incidence nor core AD pathology (Ps > 0.05). Higher PM₂.₅ was associated with higher CSF GFAP levels (*P* = 0.003). APOE4 + with higher levels of PM₂.₅ exposure had higher CSF levels of tTau (*P* = 0.01), pTau₁₈₁ (*P* = 0.01) and neurogranin (*P* = 0.02). These relationships were not mediated by neuroinflammation (Ps > 0.05). In this AD-risk enriched cohort, AP was not associated with MCI/AD incidence. However, higher PM₂.₅ exposure was associated with astrocytic activation, and in APOE4+, AD pathology, neurodegeneration, and synaptic dysfunction. Our findings suggest AP as an environmental risk factor contributing to AD-relevant pathology, particularly among genetically at-risk individuals.

## Highlights

Long-term O₃ and PM₂.₅ exposures are not associated with MCI/AD risk in a cognitively unimpaired, middle-aged and older cohort enriched for AD risk based on parental history.

Higher long-term PM₂.₅ exposure is associated with higher levels of CSF GFAP, a marker of astrocyte reactivity.

In *APOE* ε4 allele carriers, higher long-term PM₂.₅ exposure is associated with higher levels of CSF pTau₁₈₁, tTau and neurogranin.

CSFbiomarkers of neuroinflammation did not mediate the relationships between PM₂.₅ exposure and AD-relevant pathology.

Results highlight gene–environment interplay in early AD pathophysiology.

## Introduction

Alzheimer’s disease (AD) is a progressive neurodegenerative disease of complex etiology that likely involves multiple risk genes and environmental factors (Migliore & Coppede, 2022). To date, age is the strongest known risk factor for AD (Alzheimer 2024) while the ε4 allele of the apolipoprotein E*(APOE)* gene is the strongest known genetic risk factor for late-onset AD (Corder et al. 1993; Farrer et al. 1997; Saunders et al. 1993). Carrying one ε4 allele increases the risk by 20%, whereas carrying two ε4 alleles increases the risk by 90% (Corder et al. 1993). Given that AD is a multifactorial disease, efforts are focused on interactions between genetic and potentially modifiable risk factors in hopes of forestalling or mitigating disease incidence or progression, and societal burden associated with it.

Air pollution (AP), a potentially modifiable environmental risk factor, is linked to general health and 7 million (11.6%) premature deaths globally every year (World Health Organization, 2002). AP is also associated with mild cognitive impairment (MCI;Krishnamoorthy et al. 2018; Peters et al. 2019), which is considered a prodrome of AD (Petersen et al., 2009). Although the literature is largely nascent we do know that reductions in AP are associated with proportional reductions in AD and dementia incidence (Carey et al. 2018, Chen et al. 2017; Letellier et al., 2021, Peters et al. 2019, Wang et al. 2022).

Ozone (O_3_) is one of the common air pollutants that has been investigated in relation to AD. Literature suggests that repeated and long-term exposure to O_3_ leads to development of neuroinflammation, which is in turn associated with AD (Singh et al., 2023). *Postmortem* studies in people of all ages, including children, link severe O_3_exposure to neuroinflammation cerebral atrophy and tau accumulation all of which are events associated with AD (Croze & Luc., 2018).

Another air pollutant that has been investigated in relation to AD is particulate matter (PM). PM is emitted from various sources and can be categorized based on its aerodynamic diameter normally ranging between 2.5 and 10 micrometers (Tao et al., 2003; Zhang et al., 2015). Acute exposure to PM_2.5_is associated with inflammation (Arias-Pérez et al., 2020; Dubowsky et al., 2006) and synaptic dysfunction (Li et al., 2022). Long-term exposure to PM_2.5_is associated with neurodegenerative diseases (Shi et al., 2021) and increased mortality (Hao et al., 2023) in adults 65 years of age or older. Given the commonality of their presence in both urban and rural areas and their associations with neurodegenerative disease, both O_3_ and PM_2.5_ warrant investigation as possible risk factors for AD.

In the present study, we examine (1) the relationships between long-term residential AP (O_3_ and PM_2.5_) exposure and a combined risk for MCI or AD incidence, and (2) whether long-term AP exposure is associated with biomarkers of core AD neuropathology in cerebrospinal fluid (CSF) and via PET, and (3) CSF biomarkers of relevance to AD (neuroinflammation, synaptic dysfunction and neurodegeneration), and (4) whether the aforementioned relationships are moderated by *APOE* ε4 status or mediated by inflammation. Based on existent literature, we hypothesize that higher long-term AP exposure is associated with (1) increased risk for MCI or AD, and (2) the deleterious biomolecular changes of significance to AD reflected in CSF biomarkers or via positron emission tomography (PET), and that (3) the deleterious relationships between higher long-term AP exposure and PET or CSF biomarkers of relevance to AD are moderated by *APOE* ε4 status, while (4) the relationships between AP exposure and AD-relevant pathology are mediated by inflammation.

## Methods

### Participants

The sample consisted of cognitively unimpaired, late middle-aged and older adults (*N* = 795; Mean_AGE_(SD) = 68.7 ± 8; 68% female) from the Wisconsin Alzheimer’s Disease Research Center and the Wisconsin Registry for Alzheimer’s Prevention (WRAP; Johnsonet al., 2018) with available data of interest, with the exception of the survival analysis sample which in addition to cognitively unimpaired participants also included individuals with both MCI (*N* = 39) or dementia-AD (*N*= 11) consensus diagnoses. The enrollment criteria have been previously published in detail (Johnson et al., 2018). Briefly participants were 40–65 years of age at study entry fluent in English with adequate visual and auditory faculties for neuropsychological testing in general good health and cognitively unimpaired (Johnson et al., 2018). The sample was enriched at enrollment for AD risk based on parental history of AD (52%) and subsequently *APOE* ε4 allele carriage (*APOE*4+; 40%) compared to the general population, with the idea that AD pathogenesis, its predictors and the tempo of progression would be illuminated compared to their counterparts devoid of the same risks. Participants return for their second visit approximately 4 years after baseline and subsequent visits occur every 2 years thereafter. Medical history is collected by self-report.

Residential address was recorded for each participant starting with the baseline visit and only those who reported living at the same address for the duration of their participation were included in the current analyses. All procedures were approved by the University of Wisconsin School of Medicine and Public Health Institutional Review Board. Written informed consent was provided by all participants prior to study participation.

### Consensus Diagnosis

Cognitive status of participants was assessed in accordance with the National Institute on Aging Alzheimer’s Association workgroup diagnostic criteria (Dubois et al., 2021). Participants were classified as “Cognitively Unimpaired” (CU), “MCI,” or “dementia-AD”. Majority of the participants (94%, *N* = 745) were CU; 5% of the participants had a diagnosis of MCI (*N* = 39) and 1% received dementia-AD (AD) diagnosis (*N*= 11). Those diagnosed as “Impaired-Other” were excluded from current analyses. Cognitive performance and functional status were evaluated at each visit; the procedures including the cognitive battery are published elsewhere (Johnson et al., 2018). Diagnosis at each visit was used for survival analyses, and only cognitively unimpaired participants were included in all other analyses.

### Air Pollution

**Fig. 1:**
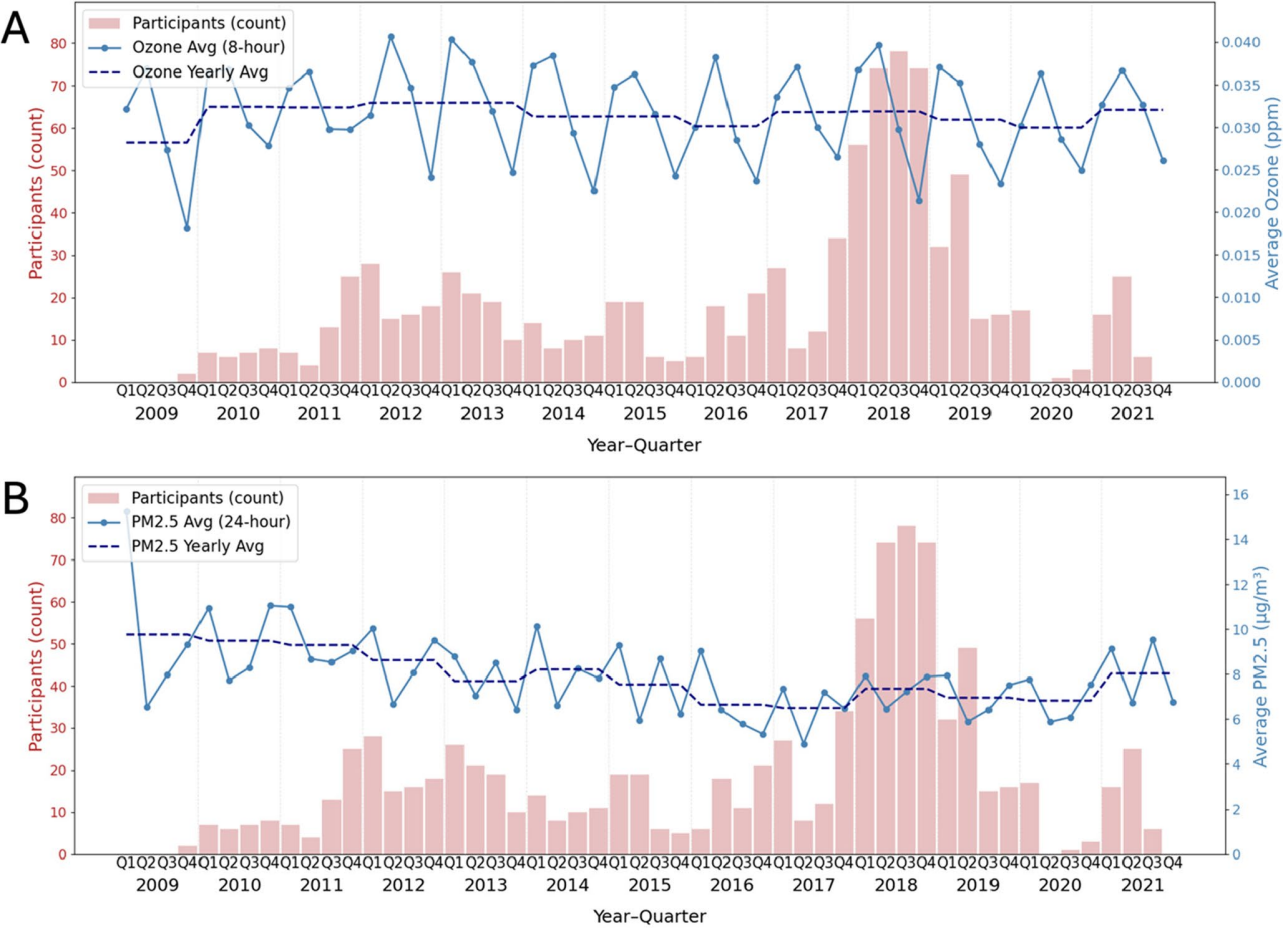
**A **O_3_ and (**B**) PM_2.5_ quarterly (solid line) and superimposed yearly (dashed line) average along with participant counts from 2009 to 2021. **A** 8-hour max daily average concentrations of the ground-level O_3_ (parts per million; ppm) per quarter (solid line) and per year (dashed line)

AP levels were determined using U.S. Environmental Protection Agency’s (EPA) annual pollution measurements based on participants’ zip codes, which should be identical between first and last visit for inclusion in this study. First and last year of participants’ recorded data for the current sample was 2009 and 2021 respectively. Thus, the AP exposure reflects the average pollutant values for each participant across follow-up (up to 13 years; Mean(SD) = 11.1(Balachandar et al. 2025) years). Figure1 depicts the AP level fluctuations per quarter year and of yearly average across the study follow-up along with the number of participants/observations for each period. The averages include all the measuring sites in Wisconsin, where 91% of our study participants reside. O_3_ is monitored hourly at each of the air quality measurement sites. From these hourly measurements, 17 different 8-hour rolling averages are calculated for each day (e.g., 7 AM-3 PM, 8 AM-4 PM,., 10 PM-6 AM; EPA, 2025). Then, the highest of these 17 averages becomes the “daily maximum 8-hour average O_3_” for that particular day. Ground-level O_3_is formed when nitrogen oxides and volatile organic compounds react in the presence of strong sunlight and high temperatures (Jiang et al., 2025). This means ozone concentrations are generally at their lowest in Q4 (fall/winter) and highest in Q2 (spring/summer). For PM_2.5_, averages are calculated over the entire day (24-hours; US. Environmental Protection Agency, 2025). PM_2.5_tends to be higher in the summer and winter months in many areas of the U.S. (Sun & Valachovic, 2025 ;Zhao et al., 2018). This is due to the use of fireplaces and increased energy consumption for heating in the winter (Q1/Q4) and also due to gases like sulfur dioxide and nitrogen oxide which undergo chemical reactions in the atmosphere to form fine particles. This process is accelerated during summertime (Q3) due to the increased sunlight and higher temperatures (Sun & Valachovic, 2025). Nonetheless, the annual averages are rather consistent across 13 years of follow-up (Fig. 1; dashed line).

### *APOE* Genotyping

DNA was isolated from whole-blood samples using the PUREGENE DNA Isolation Kit (Gentra Systems, Inc., Minneapolis, MN) and DNA concentrations were measured with Ultraviolet spectrophotometry (DU 530 Spectrophotometer, Beckman Coulter, Fullerton, CA). Genotyping for *APOE* (rs429358 and rs7412) was performed by LGC Genomics (Beverly, MA) via competitive allele-specific PCR-based KASP genotyping assays. For analyses purposes, participants were split into two groups based on genotype: carriers of at least one *APOE ε*4 allele carriers (*APOE*4+; *ε*2/*ε*4, ε*3*/*ε*4, *ε*4/*ε*4) and non-carriers (*APOE*4-; *ε*2/*ε*2, *ε*2/*ε*3, *ε*3/*ε*3).

### CSF Collection and Assays

CSF samples were collected after a 12-hour fasting period at L3-4 or L4-5 using a drip method and/or gentle extraction technique into polypropylene syringes using a Sprotte 24- or 25-gauge spinal needle. A thin needle was used to inject 1% lidocaine as a local anesthetic prior to the insertion of the Sprotte spinal needle. Approximately 22 mL of CSF was pooled, gently mixed and centrifuged at 2,000 g for 10 min. Supernatants were frozen in 0.5 milliliters aliquots in polypropylene tubes, and kept at −80 °C.

CSF samples were analyzed for phosphorylated tau (pTau_181_), Aβ_40,_ and Aβ_42_ to obtain biomarkers of **core AD pathology** (pTau_181_ and Aβ_42_/Aβ_40_), **neurodegeneration** [total tau (tTau)], **synaptic dysfunction** (neurogranin), and **neuroinflammation**[glial fibrillary acidic protein (GFAP) interleukin 6 (IL-6) S100 calcium-binding protein B (S100B) soluble triggering receptor expressed on myeloid cells 2 (sTREM2) and chitinase-3-like protein 1 (YKL-40)] using the NeuroToolKit (NTK; Roche Diagnostics International Ltd Rotkruez Switzerland). The NTK is a panel of exploratory prototype assays designed to robustly evaluate established AD biomarkers (Aβ and tau) as well as emerging markers. This toolkit enables a comprehensive characterization of AD pathology as well as a panel of synaptic axonal and glial biomarkers providing enhanced insights into the disease’s pathophysiological processes (Van Hulle et al., 2021).

### Positron Emission Tomography (PET)

Details of acquisition protocols image reconstruction processing and quantification of PET images have been previously published (Betthauser et al., 2019). Briefly, participants underwent PET imaging on either a Siemens EXACT HR + or a Siemens Biograph Horizon scanner. Parametric PET images were co-registered to 3D T1-weighted MR images [inversion time (TI)/echo time (TE)/repetition time (TR) = 450ms/3.2ms/8.2ms, flip angle = 12°, slice thickness = 1 mm no gap, field of view (FOV) = 256, matrix size = 256 × 256; voxel resolution: 1 mm×1 mm×1 mm] obtained on a 3 T GE X750 Discovery scanner with an 8- or 32‐channel phased array head coil. Aβ burden was quantified from ^11^C-Pittsburg Compound B (PiB) 70-minute dynamic PET acquisition scans by generating a global cortical distribution volume ratio (DVR) using Logan graphical analysis with cerebellum grey matter as a reference (Johnson et al., 2014). To quantify tau, standard uptake value ratios (SUVRs) using the inferior cerebellar grey matter as reference were calculated from a 20-min dynamic ^18^F-MK6240 PET acquisition 70 min post bolus injection (Betthauser et al., 2019). MK6240 SUVR was the average of all regional SUVR values corresponding toBraak stages 1 –6 (Braak & Braak, 1991).

### Statistical Analyses

Statistical analyses were performed in R Studio, version 4.2.3. Participant’s demographic characteristics were compared between three diagnostic groups (MCI, AD, and CU) via ANOVAs for parametric variables and Chi-square tests for categorical variables. We also calculated the means and standard deviations (M, SD) for each pollutant to assess average exposure levels to O₃ and PM₂.₅ across the entire sample and summarized the proportion of participants living in areas exceeding the corresponding National Ambient Air Quality Standards (NAAQS).

All models covaried for sex, age, education, race, *APOE*4 + status and parental history of AD, given their established relationships with AD risk (Alzheimer’s Association, 2024), and for years at a current residence. Age at the lumbar puncture visit served as a covariate in analyses with CSF biomarkers as outcomes and age at the PET visit date served as a covariate in analyses with PET-PiB and -MK6240 as outcomes. If multiple CSF and PET visits were recorded for a participant, we used the data for two observations that occurred closest in time. The age at the lumbar puncture visit differed from age at the PET visit for several participants; we controlled for this difference in age in our analyses.

Cox proportional hazard models examined the relationships between long-term AP exposure and combined MCI + AD risk. Regression models investigated the associations between biomarkers of core AD pathology (PET and CSF), and CSF biomarkers of neuroinflammation, neurodegeneration and synaptic dysfunction with long-term residential AP exposure. We also conducted sensitivity analyses with a sub-sample of CU participants (*N* = 48) matched to MCI + AD group based on age and sex, given that the CU group was substantially larger than the MCI + AD group. The regression models were repeated after stratifying by *APOE*4+/- status.

To test the hypothesis that inflammation mediates the relationship between AP and AD pathology, we conducted a parallel mediation analysis in which five inflammatory markers (GFAP, IL-6, S100B, sTREM2, and YKL-40) were modeled as simultaneous mediators with four core AD pathology markers (global PiB DVR, whole brain MK6240 SUVR, CSF pTau_181_, and CSF Aβ_42_/Aβ_40_ratio). This approach allowed us to examine whether each core AD biomarker uniquely contributes to the indirect effect of AP on AD pathology while controlling for potential confounders. Age sex parental history of AD and years of education were included in all models as covariates. Models were fitted using the ‘lavaan’ package (Rosseel, 2012). Maximum likelihood estimation with bias-corrected bootstrap standard errors (1,000 resamples) was applied to derive 95% confidence intervals and p‐values for all parameters. Given that multiple mediation pathways were being evaluated, *p*-values for the indirect and direct effects were corrected for multiple comparisons using the false discovery rate (FDR) procedure (Benjamini & Hochberg, 1995). Mediation analyses were repeated after stratifying by *APOE*4+/- status.

## Results

### Sample Characteristics

Characteristics of the entire sample and each diagnostic group separately are detailed in Table 1. The sample was on average 68.7 ± 8 years of age, predominately white (93%), female (68%) and college educated (Mean = 16.2 years). MCI + AD were significantly older than the CU (*P* < 0.001). There were no significant differences between the diagnostic groups with regard to sex, APOE4+ proportion, parental AD history, race, years of education, and years at residence (all *P*s > 0.05).

**Table 1:** Demographic characteristics of the entire sample and by diagnostic group

Baseline Characteristic	Entire Sample (*N* = 795)	MCI (*N* = 39)	AD (*N* = 11)	CU (*N* = 745)	*P*
Sex N (%)					0.78
Female	544 (68)	25 (64)	7 (64)	512 (69)	
Male	251 (32)	14 (36)	4 (36)	233 (31)	
Age M (SD)	68.7 (8.0)	77.5 (7.6)	75.3 (10.2)	68.1 (8.0)	< 0.001*
*APOE* N (%)					0.06
* APOE*4+	315 (40)	18 (46)	8 (73)	289 (39)	
* APOE*4-	478 (60)	21 (54)	3 (27)	454 (61)	
Unknown	2 (< 1)	0 (0)	0 (0)	2 (< 1)	
Parental History of AD N (%)					0.70
Yes	412 (52)	21 (54)	7 (64)	384 (52)	
No	203 (26)	10 (26)	1(9)	192 (26)	
Unknown	180 (23)	8 (21)	3 (27)	169 (23)	
Race N (%)					0.47
Caucasian/white	740 (93)	35 (90)	11 (100)	700 (93)	
African American/Black	42 (5)	4 (10)	0(0)	38 (5)	
Native American	7 (1)	0 (0)	0 (0)	7 (0.9)	
Asian	3 (0)	0 (0)	0 (0)	3 (0.4)	
Unknown	3 (0)	0 (0)	0 (0)	3 (0.4)	
Education (years) M (SD)	16.2 (2)	15.7 (3)	15.9 (2)	16.1 (2)	0.60
Years at Residency M (SD)	11.1 (4)	10.9 (3)	10.7 (2)	11.2 (4)	0.84
Residency in Wisconsin N (%)					0.08
Yes	725 (9)	39 (100)	11 (100)	675 (91)	
No	70 (10)	0 (0)	0 (0)	70 (9)	

* - result significant at *P* < 0.001

Abbreviations: *AD* Alzheimer’s disease, *APOE4 +* careers of at least one ε4 allele, *APOE4-* non-careers of any ε4 allele, *CU* cognitively unimpaired, *M* mean, *MCI* mild cognitive impairment, *SD* standard deviation

### Air Pollution (AP) Levels

Table 2 displays the mean concentration of each pollutant of interest across the entire sample along with the associated National Ambient Air Quality Standards (NAAQS) and the number/percentage of participants living in an area where pollutant concentrations exceed the NAASQ. Mean O_3_ concentration for the entire sample is 0.07 ppm (SD = 0.004) with 11% of the participants living in areas exceeding the NAAQS. The average PM_2.5_ concentration for the entire sample is 9.07 µg/m^3^ (SD = 0.06), with 58% of the participants living in areas with PM_2.5_ concentrations exceeding the NAAQS. We only included O_3_ and PM_2.5_ as our target pollutants given that less than 11% of the sample had available AP data for other pollutants [including carbon monoxide (CO), lead, nitrogen dioxide (NO_2_), sulfur dioxide (SO_2_), and particulate matter ≤ 10 μm (PM_10_)].

**Table 2:** Average air pollution exposure and corresponding national ambient air quality standards for the entire sample

Pollutant	Entire Sample M (SD)	Total # of participants with available data	NAAQS	Total # of participants above NAAQS
O_3_ (ppm)	0.07 (0.004)	749 (94%)	0.07 ppm	88 (12%)
PM_2.5_ (µg/m^3^)	9.07 (0.63)	494 (62%)	9 µg/m^3^	462 (94%)

Abbreviations: *M* mean, *NAAQS* National Ambient Air Quality Standards, *O3* Ozone, *PM*_*2.5*_ particulate matter ≤ 2.5 μm, *SD* standard deviation

### Associations between AP Exposure and Combined MCI + AD Risk

**Fig. 2:**
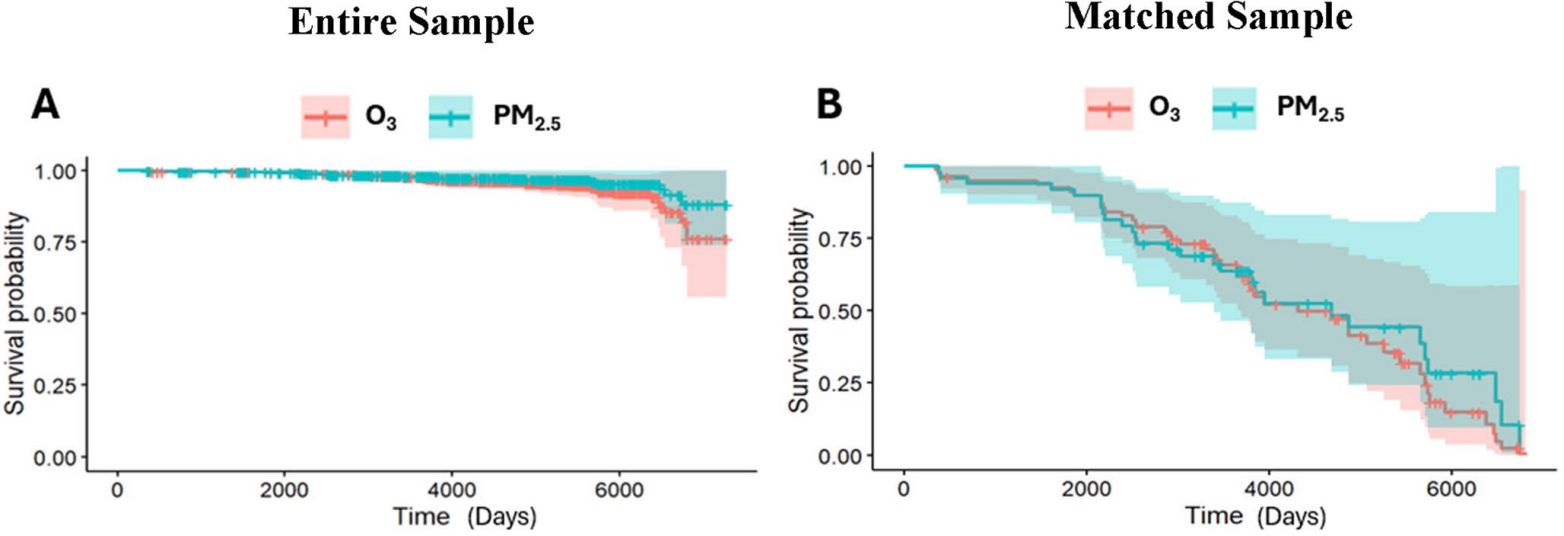
Probability of combined MCI or AD (MCI + AD) Incidence Over Time Based on AP Exposure.** A** Results for the Entire Sample (*N* = 795): no significant associations were observed between MCI + AD risk and long-term residential exposure to either O_3_ (red; *P* = 0.57) or PM_2.5_ (blue; *P* = 0.18). **B** Results from the “Matched Sample” sensitivity analyses of a subset of cognitively unimpaired participants matched to those with consensus MCI or AD diagnoses by age and sex (*N* = 96): no significant associations were observed between long-term residential exposure to either O_3_ (red; *P* = 0.91) or PM_2.5_ (blue; *P* = 0.94) and MCI + AD risk

The survival curves for O_3_ and PM_2.5_ are presented in Fig. 2A. There were no significant associations between either long-term O_3_ or PM_2.5_ exposures and MCI + AD risk (all *P*s > 0.05; Table 3). Figure 2B depicts the survival curves from sensitivity analyses, where we assessed the relationship between long-term AP exposure and MCI + AD risk in a sub-sample of CU that were matched to the MCI + AD group based on sex and age. The results remained unchanged (all *P*s > 0.05).

### Associations between AP Exposure and AD-relevant Biomarkers

There were no significant associations between long-term AP exposure and core AD neuropathology [PET (Aβ or tau); CSF (Aβ_42_/Aβ _40_, pTau_181_); all *P*s > 0.05]. Higher CSF GFAP concentrations were significantly associated with higher levels of PM_2.5_ (*P* = 0.003), but not O_3_ (*P* > 0.05). No other significant associations were observed between AP exposure and any of the biomarkers investigated (all *P*s > 0.05).

**Table 3:** Relative risk for MCI + AD based on AP exposure. (A) The results of survival analyses for the entire samples. Neither O3 nor PM2.5 were significantly associated with MCI + AD risks. (B) The results of survival analyses for the cognitively unimpaired sample (*n* = 48) matched to MCI + AD (*n* = 48) based on age and sex. Relationships between O3 or PM2.5 with MCI + AD risk remained non-significant

A. Entire Sample (n = 795)			
Pollutant	HR	CI	*P*-value
O_3_ (ppm)	3.95e-13	4.23e^−^^56^, 3.68e^+^^30^	0.57
PM_2.5_ (mg/m^3^)	0.71	0.43, 1.17	0.18
B. Matched Sample (n = 96)			
Pollutant	HR	CI	*P*-value
O_3_ (ppm)	4.26	1.25e^−^^31^, 3.99e^+^^34^	0.91
PM_2.5_ (mg/m^3^)	0.01	0.70, 1.48	0.94

All models covary for years at current address, *APOE*4 status, parental AD history, education, race, sex, and age

Abbreviations: *CI* confidence interval, *HR* hazard ratio, *O3* ozone, *PM*_*2.5*_ particulate matter ≤ 2.5 μm

### Associations between AP Exposure and AD-relevant Biomarkers after *APOE*4+/- Stratification

**Fig. 3:**
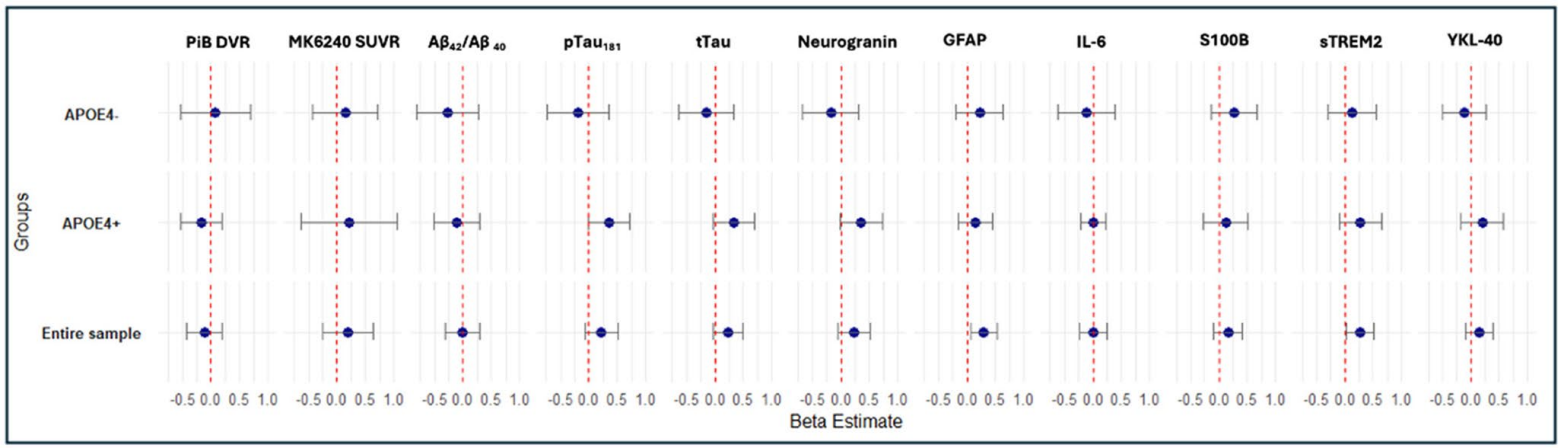
Results of regression analyses assessing the relationships between AD-relevant PET and CSF biomarkers with O3, stratified by APOE4+/- status. The relationships between O_3_ exposure and all biomarkers of interest remained non-significant after stratifying by *APOE*4 + status (all *Ps* > 0.05). * - result significant at *P* < 0.05. *Note.* All models include age, sex, parental AD history APOE4 status, and years of education as covariates. ***Abbreviations.*** Aβ = beta amyloid; AD = Alzheimer’s disease; CI = confidence interval; CSF = cerebral spinal fluid; DVR = distribution volume ratio; GFAP = glial fibrillary acidic protein; IL-6 = interleukin-6; PET = positron emission tomography; PiB = Pittsburg compound B; S100B = S100 calcium-binding protein B; SE = standard error; sTREM2 = soluble triggering receptor expressed on myeloid cells 2; SUVR = standardized uptake value ratio; pTau_181_ = phosphorylated tau at threonine 181; tTau = total tau; YKL-40 = chitinase-3-like protein 1

**Fig. 4:**
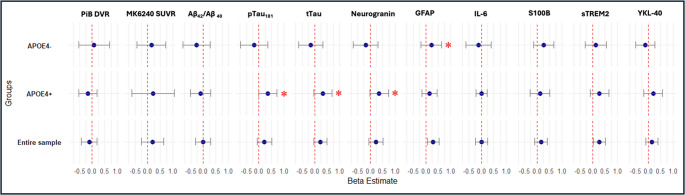
Results of regressions analyses assessing relationships between AD-relevant PET and CSF biomarkers with PM_2.5_, stratified by APOE4+/- status. *APOE*4 + compared to *APOE*4- with high long-term exposure to PM_2.5_ had significantly higher levels of pTau_181_ (*P* = 0.01), tTau (*P* = 0.01) and neurogranin (*P* = 0.02). There was a non-significant trend for the association between higher PM_2.5_ exposure and higher GFAP levels in *APOE*4+ (*P* = 0.07) and a significant association between higher PM_2.5_ exposure and GFAP levels in *APOE*4- (*P* = 0.04). * - result significant at *P* < 0.05. ***Note.*** All models include age, sex, parental AD history APOE4 status, and years of education as covariates. ***Abbreviations.*** Aβ = beta amyloid; AD = Alzheimer’s disease; CI = confidence interval; CSF = cerebral spinal fluid; DVR = distribution volume ratio; GFAP = glial fibrillary acidic protein; IL-6 = interleukin-6; PET = positron emission tomography; PiB = Pittsburg compound B; S100B = S100 calcium-binding protein B; SE = standard error; sTREM2 = soluble triggering receptor expressed on myeloid cells 2; SUVR = standardized uptake value ratio; pTau_181_ = phosphorylated tau at threonine 181; tTau = total tau; YKL-40 = chitinase-3-like protein 1

Results of *APOE-*stratified analyses for both PET and CSF biomarkers of significance to AD are presented in Figs. 3 and 4. The relationships with O_3_ exposure (Fig. 3) remain non-significant for all variables of interest after stratifying by *APOE*4 + status (all *Ps* > 0.05).

*APOE*4 + compared to *APOE*4- with high long-term exposure to PM_2.5_ (Fig. 4) had significantly higher levels of pTau_181_ (*P* = 0.01), tTau (*P* = 0.01) and neurogranin (*P* = 0.02). There was a non-significant trend for the association between higher PM_2.5_ exposure and higher GFAP levels in *APOE*4+ (*P* = 0.07) and a significant association between higher PM_2.5_ exposure and GFAP levels in *APOE*4− (*P* = 0.04).

### Inflammation Does Not Mediate the Relationship between PM_2.5_ Exposure and Core AD Pathology

The mediation analyses assessed whether the association between long-term PM_2.5_ exposure and core AD pathology is mediated by inflammation (Table 4). The direct pathways quantify the association between PM_2.5_ and each AD pathology marker separately, holding inflammatory mediators constant. The direct effects of PM_2.5_ on each inflammatory marker were also tested separately and non were significant (all *P*s > 0.05). Indirect effects represent the product of (a) the effect of PM_2.5_ on each inflammatory marker, and (b) the effect of inflammation on AD pathology. None of the pathways significantly mediated PM_2.5_’s effect on core AD pathology (all *P*s > 0.05). Total effects are the combination of direct and indirect pathways, reflecting the overall association between PM_2.5_ and each core AD pathology with the mediators and none were significant (all *P*s > 0.05). Table 5 shows the results of mediation models stratified by *APOE*4+/- status. The direct, indirect, and total effects remained non-significant (all *P*s > 0.05).

**Table 4:** Results of mediation analyses assessing if the relationship between PM_2.5_ and core AD pathology is mediated by inflammation

Pathway		*b* * (SE)*	*P*
Direct Effects	PM_2.5_ → PiB global DVR	0.004 (0.03)	0.99
	PM_2.5_ → MK6240 SUVR	0.04 (0.04)	0.66
	PM_2.5_ → CSF pTau_181_	0.10 (1.43)	0.99
	PM_2.5_ → CSF Ab_42_/Ab_40_	−0.004 (0.004)	0.63
	PM_2.5_ → GFAP	0.7 (0.5)	0.60
	PM_2.5_ → IL-6	0.11 (0.47)	0.98
	PM_2.5_ → S100B	0.003 (0.07)	0.99
	PM_2.5_ → sTREM2	0.01 (0.50)	0.99
	PM_2.5_ → YKL-40	−3.30 (11.75)	0.95
Indirect Effects	Total indirect effects	< 0.001 (0.001)	0.38
	PM_2.5_ → GFAP → core AD pathology	0.001 (0.001)	0.38
	PM_2.5_ → IL-6 → core AD pathology	< 0.001 (< 0.001)	0.93
	PM_2.5_ → S100B → core AD pathology	<−0.001 (0.001)	0.97
	PM_2.5_ → sTREM2 → core AD pathology	<−0.001 (0.001)	0.99
	PM_2.5_ → YKL-40 → core AD pathology	<−0.001 (< 0.001)	0.88
Total Effects	PiB global DVR (Direct + Indirect Effects)	0.01 (0.03)	0.89
	MK6240 SUVR (Direct + Indirect Effects)	0.04 (0.04)	0.37
	CSF pTau_181_(Direct + Indirect Effects)	0.10 (1.44)	0.95
	CSF Ab_42_/Ab_40_(Direct + Indirect Effects)	< 0.001 (< 0.001)	0.40

*P*-values are false discovery rate (FDR) adjusted

Abbreviations: *Aβ* beta amyloid, *AD* Alzheimer’s disease, *CI* confidence interval, *CSF* cerebral spinal fluid, *DVR* distribution volume ratio, *GFAP* glial fibrillary acidic protein, *IL-6* interleukin-6, *PET* positron emission tomography, *PiB* Pittsburg compound B, *S100B* S100 calcium-binding protein B, *SE* standard error, *sTREM2* soluble triggering receptor expressed on myeloid cells 2, *SUVR* standardized uptake value ratio, *pTau*_***181***_ phosphorylated tau at threonine 181, *tTau* total tau, *YKL-40* chitinase-3-like protein 1

**Table 5:** Results of mediation analyses assessing whether the relationship between PM_2.5_ and core AD pathology is mediated by inflammation; stratified by *APOE*4+/- status

	APOE4+		APOE4-	
Paths	β (SE)	*P*	β (SE)	*P*
Direct Effects				
PM_2.5_ → PiB global DVR	−0.01 (0.09)	0.97	0.02 (0.15)	0.99
PM_2.5_ → MK6240 SUVR	0.02 (0.14)	0.99	0.03 (0.07)	0.99
PM_2.5_ → CSF pTau_181_	1.83 (2.74)	0.96	−1.21 (2.56)	0.99
PM_2.5_ → CSF Aβ_42_/Aβ_40_	−0.01 (0.01)	0.96	−0.003 (0.01)	0.99
PM_2.5_ → GFAP	−0.04 (1.97)	0.99	1.12 (1.00)	0.87
PM_2.5_ → IL-6	0.33 (0.60)	0.96	−0.28 (0.86)	0.99
PM_2.5_ → S100B	−0.09 (0.11)	0.96	0.08 (0.07)	0.85
PM_2.5_ → sTREM2	−0.29 (0.79)	0.96	0.47 (0.94)	0.99
PM_2.5_ → YKL-40	−4.18 (14.74)	0.96	−2.91 (22.91)	0.99
Indirect Effects				
Total indirect effects	0.003 (0.01)	0.70	−0.001 (0.003)	0.83
PM_2.5_ → GFAP → core AD pathology	<−0.0001 (0.01)	0.99	< 0.0001 (0.001)	0.71
PM_2.5_ → IL-6 → core AD pathology	0.001 (0.003)	0.65	< 0.0001 (0.001)	0.98
PM_2.5_ → S100B → core AD pathology	0.002 (0.003)	0.57	<−0.0001 (0.001)	0.71
PM_2.5_ → sTREM2 → core AD pathology	< 0.0001 (0.003)	0.90	<−0.0001 (0.001)	0.92
PM_2.5_ → YKL-40 → core AD pathology	< 0.0001 (0.003)	0.96	<−0.0001 (0.002)	0.95
Total Effects				
PiB global DVR (Direct + Indirect Effects)	−0.01 (0.09)	0.94	0.02 (0.08)	0.81
MK6240 SUVR (Direct + Indirect Effects)	0.03 (0.14)	0.84	0.03 (0.07)	0.61
CSF pTau_181_ (Direct + Indirect Effects)	1.84 (2.74)	0.50	−1.21 (2.57)	0.64
CSF Aβ_42_/Aβ_40_ (Direct + Indirect Effects)	−0.01 (0.01)	0.54	−0.004 (0.01)	0.66

*P*-values are false discovery rate (FDR) adjusted

Abbreviations: *Aβ* beta amyloid, *AD* Alzheimer’s disease, *CI* confidence interval, *CSF* cerebral spinal fluid, *DVR* distribution volume ratio, *GFAP* glial fibrillary acidic protein, *IL-6* interleukin-6, *PET* positron emission tomography, *PiB* Pittsburg compound B, *S100B* S100 calcium-binding protein B, *SE* standard error, *sTREM2* soluble triggering receptor expressed on myeloid cells 2, *SUVR* standardized uptake value ratio, *pTau*_***181***_ phosphorylated tau at threonine 181, *tTau* total tau, *YKL-40* chitinase-3-like protein 1

## Discussion

We report no significant associations between long-term AP exposure and MCI + AD risk in our late middle-aged and older sample enriched for AD risk at enrollment. Specifically, neither O_3_ nor PM_2.5_were associated with risk for combined MCI and AD incidence and the relationships remained non-significant when the sensitivity analyses were performed with an age- and sex-matched sample of CU participants. Our findings are in contrast with both our hypothesis and the existing literature which generally reports a relationship between long-term AP exposure and AD risk (Chen et al., 2017; He et al., 2022; Shi et al., 2021; Sullivan et al., 2021).

The absence of significant findings in the present study may be due to a relatively low number of participants with MCI and AD and subsequently low statistical power to detect associations between disease incidence or AD-relevant biomarkers and AP. Sensitivity analyses which restricted the much larger CU sample to match those with MCI or AD diagnosis in age and sex confirmed the lack of significant findings. Still it is of note that the existing studies included 1000 + participants with AD diagnosis (Chen et al., Shi et al., 2021; Sullivan et al., 2021) compared to a total of 48 participants with a combined MCI and AD diagnoses in our current sample. Moreover, participants in the current study had similar levels of AP exposure - most reside in the state of Wisconsin where AP appears rather uniform across the state. This lack of variability in long-term residential AP exposure across our sample is likely to have contributed to the null findings. Limitations notwithstanding, WRAP remains a valuable longitudinal dataset for investigating AD risk, as it recruits individuals with parental history of AD and higher genetic risk of developing AD than the general population. Future studies should also consider integrating higher resolution of AP data for close areas in Wisconsin. Integrating more fine-grained analysis of AP with WRAP’s rich prospective biomarker and cognitive data would allow for examination of the role of the duration and intensity of AP exposure on AD trajectories, particularly among at-risk individuals.

We also report no significant associations between long-term AP exposure and core AD biomarkers (PET or CSF) which again is in opposition with the existing literature reporting an association between long-term AP exposure and higher levels of Aβ deposition in ALFA+ cohort of cognitively unimpaired adults at risk for AD (Alemany et al., 2021). There are several notable differences between this and our current study that may have contributed to disparate results. We used participant’s current residential zip code and 2009–2021 EPA-based annual air pollution while Alemany and colleagues (2021) applied Land Use Regression model from 2009 to estimate residential exposure to air pollutants. Moreover, some of the significant findings in their study were in relation to nitrogen dioxide (NO_2_) and particulate matter PM_10_, which we did not investigate. Furthermore, Alemany and colleagues (2021) required participants to have resided in the same residence for at least previous 3 years and all resided in the city of Barcelona, while we required that the participants have lived in the same residence over the period of up to 13 years since their enrollment in WRAP. It is also of note that Europe has pollutant profiles defined differently from the United States, that is a problem with using a proxy that does not represent accurately exposures to the smallest PM: UFPM and nanoparticles (Europeans are now doing such measurements).

When we extend the analyses to biomarkers of neuroinflammation, neurodegeneration, and synaptic dysfunction, however, we find that higher CSF glial fibrillary acidic protein (GFAP) levels are significantly associated with higher PM_2.5_exposure which is in line with the existing literature (Balachandar et al., 2025 ;Song et al., 2022). GFAP is predominantly expressed in astrocytes (Bongcam-Rudloff et al., 1991 ) and thus elevated GFAP expression in CSF is widely used as a biomarker of astrocyte reactivity and glial injury (Nielsen et al., 2002 ). It is postulated that PM_2.5_would first induce inflammation which in turn would activate astrocytes (Balachandar et al., 2025). Several mechanisms underlying glial activation in response to PM2.5 exposure have been identified; they primarily involve oxidative stress direct particle contact systemic inflammation and the release of inflammatory mediators (Kang et al., 2021; Li et al., 2022). These pathways lead to the activation of microglia and astrocytes causing neuroinflammation and neurotoxicity that can contribute to neurodegenerative disorders (Nunez et al., 2022; Thiankhaw et al., 2022).

After stratifying the analyses based on *APOE*4+/- status, we did observe the hypothesized relationships between CSF biomarkers of core AD neuropathology (pTau_181_), neurodegeneration (tTau) and synaptic dysfunction (neurogranin) in relation to PM_2.5_ exposure, with higher CSF concentrations present in those at genetic risk for AD (*APOE*4+) who are also experiencing higher levels of AP exposure. CSF GFAP levels were positively correlated with PM_2.5_, but only in *APOE*-. *APOE*4 + individuals often have higher baseline levels of CSF GFAP (Stocker et al., 2025 ), which can show up as a more robust relationships with AP in *APOE*- individuals. Indeed, we observed numerically higher CSF GFAP levels in *APOE*4 + with higher PM_2.5_ exposure (*p* = 0.07). Thus, although statistically non-significant, *APOE*4 + individuals seem to have a positive relationship between PM_2.5_ and CSF GFAP, despite its possible ceiling effect.

In animal models, E4FAD transgenic mice exposed to nanoscale urban particulate matter show 2.8-fold greater increase in cortical amyloid plaque load compared to E3FAD controls despite identical exposure, suggesting that being an ε4 carrier may drastically lower the threshold for PM _2.5_-induced neurodegeneration (Cacciottolo et al., 2017). Moreover, *APOE*4 knock-in mice treated with repeated low-dose Lipopolysaccharide injections which model environmental inflammation had significant dendritic spine loss even in the absence of significant changes in astrocyte or microglia numbers indicating that ε4 allele may heighten synaptic vulnerability (Ganesan et al., 2024). Human epidemiology studies also suggest that *APOE*4 + individuals exposed to urban PM and ultrafine particles exhibit early AD pathology and neuroinflammatory responses (Calderón‑Garcidueñas et al. 2023) worse cognition (Hsiao et al., 2023 ) and lower hippocampal volumes (Popov et al., 2024). We report a similar pattern of results with PM_2.5_ classified based on U.S. standards, while all the aforementioned studies were conducted outside of the United States and have different standards for classifying air pollutants. Overall, our findings suggest that being a carrier of at least one ε4 allele of the *APOE* gene is associate with greater vulnerability to not only genetic but also environmental risk factors known to exacerbate core AD neuropathology, neurodegeneration and neuroinflammation.

No prior study to date has investigated whether the association between AP exposure and AD pathology is mediated by neuroinflammatory mechanisms. The results of our mediation analyses suggest that there is a direct relationship between inflammatory markers and core AD pathology but no significant mediation effects of neuroinflammation on the relationship between AP exposure and core AD pathology. Still the direct relationship between neuroinflammation and core AD pathology is worthy of further investigation given that the literature generally supports a significant relationship between inflammation and AP (Block & Calderón-Garcidueñas, 2009 ; Jayaraj et al., 2017 ) and the limitations of the current study, namely the small number of participants with MCI or AD diagnoses and the lack variability in AP exposure.

Also, AP values were based on participant’s home address and data related to workplace or proximity to highways were not available. Nonetheless, the AP exposure in our study spanned up to 13 years, from enrollment to the most recent available WRAP visit and study records were carefully examined for a potential address change. Moreover, though the current study did not limit where in the United States participants lived, 91% of the participants resided in Wisconsin where O_3_ exposure levels were relatively low across the state. The O_3_ and PM_2.5_ were monitored and collected according to the U.S. EPA standards, potentially affecting the generalizability of our findings to those of studies conducted outside of the U.S., yet most report a similar pattern of results Third, the current study has a small sample size of participants with MCI and AD diagnosis. Although the sensitivity analyses showed similar results, careful interpretation of our results within the context of its limitations is required.

Overall, our results suggest that being a carrier of one or both ε4 alleles of the *APOE* gene may uniquely increase sensitivity to environmental factors, such as long-term AP exposure, which is known to exacerbate core AD pathology, neurodegeneration, and synaptic dysfunction. Moreover, studies with larger samples are needed to better understand the risk associated not with carrying one vs. both risk (ε4) but also the protective (ε2) alleles. Nonetheless, our findings highlight the need for consideration of air quality when assessing AD risk, especially for those with genetic predisposition for the disease. Further, our findings suggest that reducing long-term AP exposure could be a potentially viable practical intervention aimed at slowing the disease onset or progression in *APOE*4+. As urbanization intensifies and percentage of older population increases globally, management of AP levels may become a crucial step in both risk assessment and as preventive strategy for AD.

## Data Availability

No datasets were generated or analysed during the current study.
